# Community Member's Perspective: Serving on an Institutional Review Board

**DOI:** 10.31486/toj.19.0099

**Published:** 2020

**Authors:** Brian T. Butcher

**Affiliations:** Retired Professor of Medicine

I joined the Ochsner institutional review board (IRB) following a discussion with my physician who was, at that time, the head of the board. During my 50+-year career as a research scientist, I had served as a member of IRBs for 25 years, representing the needs of medical research institutional scientists, but I also understood that a major role of the IRB was to ensure that the safety and interests of patients were represented. Therefore, I took the opportunity to offer my services to the Ochsner IRB as a community member representing the public. Unlike many non-Ochsner IRB community members, I could offer a strong scientific background in a wide variety of fields: immunology, microbiology, multiple drug resistance, epidemiology, and gene therapy. In many of these areas, I had actually worked with human subjects and had submitted proposals to the IRBs at various research institutions, including Ochsner where I was located for a time following Hurricane Katrina.

Performing research with human subjects is a privilege that demands special care from physicians and clinical researchers. Equally, such research requires special attention from board members to ensure that minimal harm can come to patient volunteers.

Why did I join the IRB as a community member? My principal reasons were the camaraderie associated with serving and the feeling of doing something very important. Much of one's effort as a board member involves attending board meetings and discussing research proposals. Prior to attending meetings, board members expend a great deal of effort reviewing proposals and consent forms. Being a board member is not easy service; the proposals to be reviewed are often difficult to understand and very complex because they are written for specialized medical areas. Consent forms are somewhat easier to review, as they must be written in simple language that is easy for volunteer subjects to understand and thus less taxing for the board members reviewing them.

At Ochsner, the IRB office usually makes the upcoming agenda available to board members approximately one week prior to the meeting, so board members know what submissions they are assigned to review. Board members are responsible for reviewing their assigned submissions in depth and reporting the results of their reviews to the full board at the monthly meeting. We review initial submissions, renewals, and ongoing studies. In addition to reviewing the applications for which one has responsibility, good practice is to get a basic overview of all the other submissions on the agenda. Meetings generally last 2 to 3 hours.

The Ochsner IRB has an excellent system. For each new protocol, the senior researcher is expected to attend the meeting and give a summary of the proposed study. Members of the committee have the chance to ask questions and express any concerns. Following the presentation and question session, the committee discusses the application. First, the board member who reviewed the proposed research will share any concerns and whether the senior researcher addressed them. Members of the board will ask the reviewer questions and share any concerns they have about the study. Then the board member who reviewed the informed consent form will discuss his/her review; the proposal reviewer often complements this discussion.

When a member is assigned to review an informed consent form, several aspects of the form must be considered. First, the form must be written in an easily understandable way. Ideally, language should be at an 8th grade reading level or below, and all technical terms should be simplified. Grammar and syntax must not impede understanding. The form must clearly state that the volunteer is enrolling in a research study and that the study may not provide any medical benefits. Informed consent forms must spell out possible positive and negative risks, including possible death, and all of this information must be in lay language.

Another important role of reviewers is to determine that potential volunteers are not subjected to undue inducement. Undue inducement can be difficult to evaluate: what might constitute minor financial compensation for some can be a major inducement for others. For example, $50 can be a significant amount when offered to a struggling student but not to a successful businessman or businesswoman.

IRB members must be prepared to attend meetings each month to discuss and vote on proposals. Ochsner also schedules quarterly meetings at which staff update IRB members and discuss new federal and state rules and regulations.

Serving as a member of the IRB is an honor, and board members should be aware of their important role in evaluating proposals and protecting the health and safety of human subjects.

